# Making Sense of Corporate Social Responsibility and Work

**DOI:** 10.3389/fpsyg.2016.00443

**Published:** 2016-03-24

**Authors:** Ami N. Seivwright, Kerrie L. Unsworth

**Affiliations:** ^1^Centre for Social Impact, Business School, University of Western Australia, CrawleyWA, Australia; ^2^Organizational Behaviour, Leeds University Business School, University of LeedsLeeds, UK

**Keywords:** employee behavior, corporate social responsibility, meaningfulness, job design, organizational behavior

## Abstract

Employees can be a driving force behind organizational corporate social responsibility (CSR) efforts, yet the vast majority of literature has focused on firm-level understanding and implementation of CSR. Recent literature that explores the relationship between employees and CSR has not investigated how employees conceive of their role in CSR. We propose that in order to understand the factors that affect employee engagement in CSR, we must first understand how employees conceptualize the phenomenon of CSR and how that conceptualisation fits into their work. Our exploratory, inductive study interviews two cohorts of employees, one in a not for profit and the other in a corporate organization, revealing stark contrasts in how the different cohorts conceptualize and engage in CSR, particularly with regards to how CSR contributes to meaningfulness at work. Implications for organizations are discussed.

## Introduction

In academia and in the C-suite of organizations, we have an understanding of what corporate social responsibility (CSR) is and what needs to be done to achieve CSR strategies; but what do employees think their involvement in CSR is and how do they make sense of this construct, particularly when it comes to how it fits into their work? The construct of CSR is well-established in the literature, with much effort dedicated to defining and developing it (e.g., Davis, 1960; Eells and Walton, 1961; Carroll, 1979; Wartick and Cochran, 1985), investigating how best to focus firm-level efforts (e.g., Wood, 1991; Garriga and Melé, 2004), and considering the outcomes of CSR, including employee-level outcomes such as satisfaction, commitment and performance (e.g., Turban and Greening, 1997; Gond and Herrbach, 2006; Lee et al., 2013). However, much CSR needs to be enacted by employees in its implementation and little emphasis has been placed on this role; that is, looking at how employees contribute to CSR rather than just how they are affected by it. This represents a serious gap in our understanding of CSR as it is employees who are often responsible for enacting an organization’s CSR policy and strategy, yet we do not know what they understand CSR to be.

The CSR literature emphasizes that multiple stakeholders must be engaged to optimize the outcomes of CSR efforts (Meznar et al., 1990; Knox and Maklan, 2004; McWilliams et al., 2006). Accordingly, the role of micro, meso and macro level stakeholders has been explored through conceptual frameworks (e.g., Aguilera et al., 2007; Rupp et al., 2011), multilevel models (e.g., Wood, 1991) and extensive literature reviews (e.g., Aguinis, 2011; Aguinis and Glavas, 2012). These explorations highlight that research on the micro level, particularly at the employee level, is severely lacking. Some authors conceptualize leaders and managers as the micro level of CSR, noting their importance as they are often responsible for selecting implementing CSR strategy (Wood, 1991; Basu and Palazzo, 2008; Jiraporn and Chintrakarn, 2013; Okoye, 2013). However, this does not allow for a comprehensive understanding of CSR as it suggests that leaders are the final actors in implementing CSR, when in reality, it is up to employees to enact the strategy set out by the organization and supported by its leaders. As a result, while firms accept that they must engage stakeholders at all levels in order to effectively meet their responsibilities to their operating environment, the connection between employees and CSR, and the impact of employee CSR effort on the firm – both generally and in terms of corporate social performance, is not clearly established, meaning CSR in reality is often just the production of expensive reports for the purpose of compliance (Knox and Maklan, 2004). We believe that understanding how employees conceptualize CSR and, in turn, how this conceptualization affects their CSR behavior is critical to establishing how employees fit into the CSR picture.

Following Carroll (1991, 1999) and Dahlsrud (2008), we define CSR as being based on stakeholder needs, being financially sustainable, including the environmental dimension, and either voluntary or economically, legally or ethically mandated. Thus, we suggest that CSR can come from both corporate organizations whose remit is outside CSR or from not-for-profits who are driven to engage in CSR for other reasons (see Aguinis and Glavas, 2013 for a discussion on embedded versus peripheral CSR). We consider eCSR to be the employees’ efforts to then enact the organization’s CSR strategy (or, to substitute with personal behavior if the organization’s CSR strategy is deemed inadequate); more specifically, we define it as employee behavior, engaged in at work, with the intention of benefiting society or the environment. This is different to other individual level constructs because of the lack of direct feedback. For example, pro-social behaviors are directed at actors within the organization or known to the employee, and as a result the employee can expect a level of feedback, reciprocation or reward. On the other hand, eCSR is intended to deliver benefit external to the organization (the environment or broader society), and often the employee will never be able to know the true outcome of their behavior, let alone reap professional benefit from it. While there are certainly some *cases* of pro-social behavior (or altruism, extra-role behavior, interpersonal helping, etc.) that could also be considered eCSR, these constructs do not encompass our above definition. We therefore believe that the exploration of the eCSR construct, which fully encompasses how *employees* make sense of and engage in CSR activities, irrespective of their organization’s position, is important to building the micro-foundations of CSR.

When considering employees and CSR, some recent literature has explored employee outcomes of CSR, such as improvements in employee performance, behavior and attitudes resulting from participating in CSR (Glavas and Piderit, 2009; Jones, 2010; Lin et al., 2010; Mueller et al., 2012). This research consistently finds that CSR is positively received by employees in terms of traditional outcomes, yet very few studies have explored the other side, that is, what CSR means to employees and how employees contribute to it through their behavior. As exceptions, Hemingway (2005) and Rodrigo and Arenas (2008) theoretically conceptualize categories of employee attitudes toward CSR but have not explored whether these categories emerge in organizations nor whether they are comprehensive. Other research has looked at antecedents of eCSR: Bingham et al. (2013) propose that employees are more likely to participate (in either an affiliative or actionable way) in organization-sponsored causes if they are committed to the cause and feel the organization is genuinely committed to the cause, and in their longitudinal study, Smith (2013) found that strong organizational philanthropic identity and efforts, over time, increased employees’ charitable attitudes and behavior. Chen and Hung-Baesecke (2014) surveyed employees on their current engagement and future intention to engage in 23 activities that the organization offered and linked this to leader behavior.

Yet these studies made assumptions about the conceptualization of eCSR; we argue that the construct is too new for us to truly understand it from afar at the moment. Instead, inductive studies need to be conducted to determine how employees conceive of their role in CSR. One study that did take an exploratory look at employee engagement in CSR is Slack et al. (2015). In a case study in a single organization, they found differences in the level of eCSR amongst employees as well as differences in whether employees engaged in organizational or personal engagement in CSR outside of the workplace. This latter surprising finding demonstrates the importance of taking an inductive approach to eCSR at this stage. However, a single case study is only a start to this line of inquiry. We argue that more inductive work is required in alternative types of organizations as this may affect the conceptualization, for example, whether employees in organizations with CSR embedded into their core strategy conceptualize and engage in CSR differently than employees whose organization enacts CSR as a peripheral activity (Aguinis and Glavas, 2013). In particular, it is likely that employees in non-profit organizations (which still need to be financially sustainable, even if not financially profitable) will view their engagement in CSR differently to employees in profit-making companies (such as the one studied by Slack et al., 2015).

We believe that it is vital that we understand how employees make sense of CSR and conceptualize it before we can truly begin to identify factors that affect their engagement in CSR activities. For example, if employees perceive CSR to be an opportunity then it is likely that intrinsic motivation is at play and constraints and barriers will be the most influential factors affecting engagement, but if employees perceive CSR to be an obligation then reward will be more influential to increase the extrinsic motivation required to engage. Given that the factors that affect a particular behavior will differ depending on how the individual perceives that behavior, it is important that we execute this first step in the program of eCSR research as comprehensively as possible.

Therefore, while most of the micro-CSR literature to date explores either how employees’ conceptualization of the organization’s CSR affect traditional employee outcomes or what affects such conceptualizations, this study sets out to explore how employees in profit-driven and non-profit organizations actually conceptualize and engage in CSR. We feel that this is an important step as, despite the recognized importance of eCSR, there is a lack of inductively based conceptualizations of the full scope of behaviors that employees engage in with socially responsible intentions. Our research question, therefore, was, “How do employees engage in the enactment of CSR and how do they make sense of how it fits in with their work?” We conducted an inductive study with employees from two organizations, theoretically sampled to ensure we had both a not-for-profit organization and a corporate organization.

## Materials and Methods

We used the qualitative research tool of interviewing for data collection. Morgan and Smircich (1980, p. 491) state that the appropriateness of qualitative methodology in social research “derives from the nature of the social phenomena to be explored,” with Huberman and Miles (2002) specifying that qualitative research is essential to understanding individual perceptions and social interactions. As there is no existing theoretical or empirical conceptualization of employee CSR behavior or even an understanding as to how employees conceptualize this themselves, the research is exploratory to ensure that our eventual conceptualization of this type of behavior is reflective of how it is enacted and experienced in real life.

Qualitative interviews were conducted with 32 employees at two different organizations. Organization One is a prominent faith-based not-for-profit organization that has a operations worldwide. The workforce of Organization One consists primarily of volunteers, however, as this research focuses on employee conceptualization, our sample was 17 paid employees in professional positions across various areas of the organization. Nine were female and eight were male. Twelve participants worked in the headquarters of the organization and occupied a range of positions from management, call center operation, building management, accounting and finance, training and development, and counseling. Three worked at an adult mental health facility in managerial and clinical positions and two worked in a youth homelessness center in managerial and counseling roles. Both facilities are run by the organization. All of the locations were within the metropolitan area of Perth, Western Australia. Employees were recruited via e-mail initiated by the CEO of the organization inviting employees to contact the researcher if they were interested in participating. Once the participants contacted the authors, we found a mutually beneficial time and one author traveled to their workplace and conducted the interviews. These interviews were conducted individually (one per day) at the convenience of the participants over the first half of 2014.

Organization Two is a large organization in the banking and finance industry. The sample consisted of 15 employees, also employed in a range of areas across the business, including information technology, marketing, management, and strategic planning. Nine of these participants were female, six were male. Eleven out of fifteen reported engaging in corporate volunteering during their tenure with the organization, four had not. The interviews were conducted over 2 days in November 2014 in the headquarters of Organization Two. A member within the organization recruited and arranged appointments for participants to be interviewed, and the organization offered a 5AUD donation to charity on behalf of each participating employee as an inducement. This was organized independently of the researchers.

As emphasized in the introduction to this article, we believe that the micro-level of CSR research concerning employees and CSR is too new for us to understand well-enough to have reliable assumptions that we can build our research upon. Therefore, it was critical to us that our study was inductive and enabled us to capture the full scope of behaviors that employees believe constitute CSR. Therefore, the interview schedule began simply by describing what we believe to be the core characteristics of employee CSR – behavior engaged in at or through work with the intention of benefiting the environment or society; and asking the participants if they could recall any instances of engaging in such behavior over the last 12 months. This was the primary source of data that gave us insight into how employees thought about their behavior at work and the benefits they believed their actions offered to the environment or society. Rather than simply asking if they engage in a specific behavior (e.g., donating to charity through their workplace), we left the question open to gain insight into a broader range of actions that employees engage in with a socially responsible intention.

Also in line with the inductive approach, the interview was conducted in a semi-structured manner that adapted to each interviewee. When a participant listed more than one behavior they engage in at the initial prompt, the interviewer noted down all of the behaviors mentioned and asked the participant to discuss each one in detail, one at a time. For each behavior, the participant was asked what the behavior involves, why they do it, if it’s facilitated and/or encouraged by the organization and/or their supervisor, what obstacles they face in engaging, and how engaging makes them feel. If the participant only listed one behavior, the interviewer prompted again, “are there any other things you do at work to benefit the environment or society?,” followed by “what sort of initiatives does the organization encourage employees to participate in?” and “do you participate?” “why/why not” etc., then “is there anything you do personally at work, even small things that nobody else may know about, to benefit the environment or society?,” investigating each behavior in depth.

The research was carried out in accordance with the recommendations and approval of The University of Western Australia’s Ethics. All participants were verbally informed that they would not be personally identified at any stage of the research, they did not have to answer any questions they were uncomfortable with and could withdraw at any time. They were given a participant information form outlining the purpose of the research and the data procedures, as well as the contact details for both authors should they have any questions or concerns at any time. Adequate time was provided prior to the commencement of the interview for the participant to read the information form, and the interviewer verbally checked if they had any questions prior to commencement, and if they were happy to proceed. Each participant signed a consent form confirming that they had been provided with the information form and had read it, and were happy to participate in the interview. Prior to starting the recording, the interviewer confirmed again that the participant was comfortable with me recording the interview, and the recording device was kept in clear view and reach of the participant at all times. Neither author has had any communication from participants after the interviews.

Transcripts of the interviews were analyzed in the R Qualitative Data Analysis (RQDA) package by the interviewing author using a grounded theory approach, which is best suited to this study as we seek not just to describe the findings within the data, but to build theory through the data (Strauss and Corbin, 1990). First, open coding, which involves line by line analysis of the data and assigning each line a theoretical concept that is relevant to the phenomena being explored, was conducted (Glaser, 1978; Strauss and Corbin, 1990). Then, axial coding, grouping the open codes through empirically grounded links occurred, followed by selective coding, which involves integrating axial codes into a coherent theoretical framework that answers the research question (Strauss and Corbin, 1990). In terms of reliability, the author that conducted the interviews wrote notes after each interview. These notes were put aside and the same author conducted the open coding. A researcher completely separate to the study checked the codes with 80% agreement. The authors then discussed the codes and statements that were contested and decided together whether to keep each code, merge with other existing codes, or recode it completely. The interviewing author then presented initial findings to the other author, who interrogated the data for negative cases and alternative explanations. Only findings that both authors were confident with regards to the evidence available are presented.

## Results

To set the stage for the results around the microfoundations of CSR, it is important to outline how CSR was constructed at the organizational level for the two organizations we studied. The mission of the NFP organization was centered around social responsibility, with a particular focus on social issues such as poverty, homelessness, financial hardship and mental health. The organizational structure was unique in that most of the operations were dependent on volunteers, so participants reported that the CSR context was shaped by all staff activities contributing to an overall socially responsible mission, and that the mission was primarily enacted by volunteers not employed by the organization. This is reflected in the annual report of the organization, which identifies employed staff as either providing direct service such as counseling and medical care, or operational support such as finance and human resources. To support the central operations, the organization hosted an annual fundraiser that staff were encouraged to attend and raise money for, and, according to middle management, staff were encouraged but not expected or required to engage in volunteering outside of work with the organization. Interestingly, although the social aspect of CSR was strongly evident, the organization reported no environmental policies or procedures in the annual report.

The corporate organization reported a more ‘traditional’ approach, stating in their annual report that they engage in CSR by focusing primarily on delivering value to stakeholders while being mindful of the environmental, social and economic impacts of its operations. However, the organization had an excellent reputation amongst national volunteering organizations as an industry leader in the voluntary sphere of CSR. The major focus for staff engagement was volunteering, with the employees having two paid days off available for volunteering at particular charities through the organization’s programs. In addition, the organization matched individual employee donations and allowed employees to apply for small (1000AUD) grants for community organizations that they wanted to support. Although the organization’s parent company was involved in federally mandated environmental reporting, specifically National Greenhouse and Energy Reporting, it did not appear to be a major focus, with the annual report stating that the organization is not subject to further legislated environmental regulation.

It seems, therefore, that the NFP organization had an embedded CSR strategy that came from the core of its business (cf. Aguinis and Glavas, 2013). Notably, anything that was outside this core, such as environmental sustainability, was not addressed, presumably because of the embedded strategy. On the other hand, the corporate organization had a peripheral CSR strategy where CSR activities were outside the main core strategy of the business. CSR covered both social and environmental aspects, perhaps because the “freedom” of the peripheral strategy meant that a wider variety of CSR activities could be undertaken, however, there was still a very clear focus on social and community responsibilities rather than environmental.

### Within-Role versus Extra-Role CSR

This investigation of the organizational level CSR approach occurred before we conducted the interviews and initial data analysis so as to avoid potential biases (Strauss and Corbin, 1990). Nonetheless, we found that this differentiation between embedded and peripheral CSR emerged spontaneously in the employees’ interviews. Generally, perceptions of CSR were different depending on whether the person came from the NFP or from the corporate organization. Broadly, most NFP employees perceived that their actual core job was CSR behavior; indeed, 11 out of the 17 participants responded to our initial question of “We’re looking to discuss things you do at work with the intention of benefiting the environment and/or society. Can you think of anything that you’ve done at work that would fit that description?” with comments that that was just part of their job and two others discussed this in relation to later questions. For example, they made statements such as “my whole role I suppose does that” (NFP16), or “well working for [NFP X], that automatically means you’re working to help others in society” (NFP1), or “the main thing that I do is choose the type of work that I do. So actually my career and what I’ve actively chosen is to work in the community sector” (NFP17). A statement that captures the nature of the within-role rather than extra-role nature of CSR came from NFP14: “Well there’s nothing in any policy of ours that says every staff member will have 2 days of community work. I suppose then one would argue that we are actually doing community work because you’re doing community work every day in coming to work, because ultimately that’s what your whole job’s based around.”

On the other hand, most corporate employees responded with statements about their participation in the organizationally supported volunteering, such as “...that’s a bit tricky ‘cause I put my hand up [to be interviewed] because I haven’t done any volunteering over the past 12 months, so I don’t know if there’s anything I’ve done that has explicitly benefited the environment or society” (CORP3), “So we do occasionally have volunteering opportunities come up. So I did one recently where we got the opportunity for almost everyone in my broader division to do a volunteering day” (CORP5), or “Well, probably would be the volunteering. So volunteering through the [organization]” (CORP11), “So in my day job, I don’t – day-to-day, I don’t think there’s much that directly impacts that. But through volunteering at [organization] and I get involved with opportunities to volunteer for events or other community engagement stuff” (CORP8).

In other words, it appears that CSR is conceptualized by the majority of NFP employees within this sample as something that implicitly emerges from their job and that it is within-role rather than extra-role behavior. On the other hand, the majority of corporate employees sampled viewed CSR as something external to their job, that is an explicitly CSR and something that constitutes an extra-role behavior. As we argue in the discussion, this is an important contribution because the factors that lead to higher levels of within-role behavior are different to those which lead to higher levels of extra-role behavior.

Interestingly, there were also different perceptions of the motives behind the organization’s CSR strategy in the corporate organization, but not in the NFP. When talking about the corporate organization’s CSR strategy, some people took a more cynical perspective that the CSR was for reputation-building and lip-service, even while acknowledging the positive nature of the activities. For example, CORP11 said, “I think it’s a good thing [for me] personally and I think it’s also a good – it’s a tick for the bank as well ‘cause it’s – they can, do all the nice community side of things, and – so, look at us, we’re good,” and CORP6 said “When we first moved in here, it was all about the big green star – five star green star rating that we all gained in this building as well as other stuff. But then when we see the cleaners or some people have mentioned when they see the cleaners actually removed the bin bags they put it all together in the end.” While this did not occur often, it is interesting that it emerged at all in a sample that volunteered to talk with us about CSR. And, notably, it did not emerge in the NFP sample, possibly due to the difference between embedded and peripheral strategies (Aguinis and Glavas, 2013).

### CSR as a Means to Achieve Meaning

We found that many participants, all of whom worked for the corporate organization, viewed CSR as a way of achieving a meaningfulness that they were lacking in their job. The contrast between volunteering and day-to-day work, and particularly the difference in impact on society was noted by participants, with statements such as “I like the volunteering because it does feel that you are able to make a contribution. It might be a small contribution. It might be a big contribution. But it just feels like you are able to give something to the community” (CORP11) or “you really get a sense that there’s something a lot bigger out there than just what we’re just doing at work” (CORP15) or “I guess with all the negative publicity we’re on banks and all the rest of the financial sector, I don’t wake up, I guess, in the morning going, “Yes, I work for a bank. Am I contributing to society?” I don’t get that really. But – and again, that’s where the volunteering days help in that they, I guess, do make me feel a bit better, that – well, today I’ve actually, I’ve helped out in a very – and it – what feels to me much more kind of a way” (CORP1).

On the other hand, several of the corporate participants expressed mixed feelings about how their work impacts society, with some noting that although their job does not explicitly benefit society, it provides a basic service that society needs, and noting that the organization’s initiatives as a way to provide explicit and tangible benefits to society. This occurred when the participants had not engaged in the extra-role CSR activities, or had done them cynically, and could potentially be seen as trying to find an alternative way of creating meaning. For example, one participant stated she hadn’t had time to participate in the volunteering initiatives that the organization encourages, but said “I think indirectly, there’s a lot of work that I do that supports our society because we look after people’s money and we help them, and we help them make financial decisions and safe guard their cash and their assets and help them buy cars and homes and that type of thing” (CORP3). Similarly, though CORP10 had not participated in any of the organization’s CSR initiatives, she felt that she benefited society as her role involved determining and communicating the organization’s CSR strategy. CORP2 and CORP14 undertook CSR initiatives because “it’s a day off” and because “my whole team was going” and they both recognized that their roles didn’t benefit society; but CORP2 still said “I mean, there’s obviously the benefit that we deliver to our customers through doing what we do,” as did CORP14, stating “We develop the website. Yes. Ultimately, it’s the users – the society. Yeah. If you think those customer experience point of view, yeah, I can do something that benefits the society in that aspect. But not that of the general public.”

Thus, it appears as though, at least for our participants, that CSR was a way of achieving meaning from one’s work; when participants had a job which was already perceived as meaningful (i.e., NFP employees) then they did not feel a need to engage in CSR as a separate activity and when participants were not able or willing to engage in extra-role CSR activities, they reframed their job to identify some meaning, no matter how tenuous.

### Proactive Environmental Behavior

Although this difference between NFP and corporate employees held for the majority of cases, there were three instances of NFP employees who discussed extra-role CSR behavior rather than within-role CSR. All three participants (NFP15, 3 and 2) mentioned environmental initiatives that they had proactively implemented in the workplaces. Participant 15 had introduced a recyclables bin in the office kitchen to collect aluminum cans and was doing the legwork (cleaning and dropping off the cans to the collection center) around that, NFP2 had introduced recycled copy paper, toilet paper and paper towels to their office, arranged recycling of batteries, as well as questioned senior members of the organization as to why the organization lacks a clear environmental policy, and NFP3 purchased and placed a recycling bin in the communal kitchen, switched default printing setting to grayscale, introduced recycled copy paper, removed individual printers and educated the office on reducing their printing overall.

In trying to understand this finding, we examined a number of plausible explanations. First, we examined whether it was the job-role that was at play; in other words that these three employees were office-based (and thus needing to seek meaning in extra-role CSR behavior) and the others were all counselors (who could seek meaning from within their role). However, this was not the case because although NFP15 was the Chief Financial Officer and NFP3 was a regional manager, NFP2 was a counselor, and there were 10 other office-based staff who focused on within-role CSR rather than extra-role CSR. We also examined whether it was due to age and gender; however again, there was no distinction between these three and the rest of the sample. Instead, we found two issues which may expand our understanding. These three employees expressed strong pro-environmental values. For example, NFP3 said “I just couldn’t believe that it [recycling] wasn’t happening here, I mean everybody recycles at home, you know?,” NFP2 said, “It seems to me than an organization like this should be leading the way with environmental policy, not lagging behind” and NFP15 said, “I suppose it’s just my upbringing… this does affect the environment too type of thing.” Therefore, it could be that NFP employees’ sampled felt that the pro-social/community based element of CSR was incorporated within their core job role but that their core role was not related to corporate environmental responsibility triggering extra-role CSR behavior in those who had strong environmental beliefs.

An alternative, or complementary, reason for this difference may be the relative salience of these particular extra-role CSR behaviors. These activities seemed to be top of mind for these employees not only because they were self-initiated, but also because they had experienced resistance and frustration in implementing them. NFP15 had to cease his can collecting because colleagues claimed that it was attracting ants to the kitchen, NFP2 had been e-mailing his superiors and answering employee surveys for 3–4 years to get these initiatives implemented organization-wide, and NFP3 had to repeatedly educate and correct her staff through the changes. NFP1 mentioned similar environmentally conscious behaviors, but not on the initial prompt, and she engaged in them at the suggestion of a colleague, rather than independently. In other words, it might be the method of data collection that influenced the discussion of extra-role CSR behaviors for these NFP employees but not in other NFP employees. Further research is required to unpack this further.

## Discussion

In this research we wanted to explore how employees make sense of the concept of CSR. Most of our understanding of CSR is at the level of the organization, yet it is employees who are actually engaging in the behavior. If we want to ensure that they do engage in CSR and that the CSR strategies that are set at the level of the organization actually work then we must understand, first and foremost, what employees think about CSR generally.

In a qualitative study of 32 employees in both a NFP and a corporate organization we found that there was not an homogenous view of what CSR entailed. Instead, we found that it depended on how much meaning the employee was able to extract from their core job role. Although, these findings emerged directly from the interviews and analysis without referring to the literature, this neatly mirrors Aguinis and Glavas (2013) distinction between organizational-level embedded CSR and peripheral CSR strategies. Embedded CSR is when CSR is incorporated into the organization’s core strategy while peripheral CSR are initiatives that are not directly related to the organization’s core strategy (e.g., volunteering). It can be seen that the NFP has an embedded CSR while the corporate organization has peripheral CSR. While Aguinis and Glavas (2013) discussed embedded and peripheral CSR at the organizational level, we found that the distinction occurred at the level of the employee perception and sensemaking.

Interstingly, Aguinis and Glavas (2013) also discuss meaningfulness but only how it moderates between organizational-level CSR and employee-level outcomes such as identification, psychological contracts, justice and engagement. On the other hand, we found that the level of perceived meaningfulness (either at work or in work) led to a perception of CSR as either within-role (presumably an embedded CSR strategy) or extra-role (presumably a peripheral CSR strategy). Thus, it is not just the organizational level CSR strategy itself which affects whether an employee views it as embedded or peripheral at their own level, but their perceived meaningfulness. It is likely that the employees we interviewed from the NFP also perceived higher levels of meaning from their work, as this has been seen in many reviews of meaningfulness at work (e.g., Wrzesniewski et al., 2003; Rosso et al., 2010). Supporting Aguinis and Glavas (2013) propositions, we found that a few participants from the organization with the peripheral strategy identified mismatches between the CSR strategy and the intentions behind it.

An alternative way of interpreting these findings is to see perceptions of CSR as dependent on progress toward goal completion (see e.g., Louro et al., 2007; Unsworth et al., 2013). If an employee has a goal of “creating meaning” that is achieved through his or her core job role then he/she will perceive CSR as embedded within his or her job; the goal is met through tasks that already have to be performed and no additional effort outside the role is needed. On the other hand, if that goal is not achieved in the core job role, either because the organization has a peripheral CSR strategy (e.g., the corporate organization in our sample) or because one’s overarching goal of “creating meaning” incorporates other aspects such as pro-environmental goals then additional effort is needed to fully achieve that goal and CSR becomes viewed as extra-role. So what does this mean for our understanding of microfoundations of CSR? We suggest that, first and foremost, it highlights the importance of examining individual perceptions of CSR. Even with the same organizational-level CSR strategy, people made sense of CSR in different ways, depending on the level of meaning of their work and the degree to which their “meaning” goal was met. This has a number of implications for how we try to increase employee engagement in CSR.

One consideration is the role of job characteristics in relation to employee CSR behavior. Our data indicates that people perceive and engage in CSR differently depending on how it fits with their role and whether their role creates meaningfulness, which are core elements of Hackman and Oldham’s (1975) model, yet how to integrate CSR into job design remains unexplored. Interestingly, although those in roles with embedded CSR engaged in a greater amount of CSR by default, those with peripheral CSR tended to engage in and be concerned with a broader range of behavior. This raises interesting questions about how to design a role with embedded CSR without limiting employees’ willingness and ability to engage in a broader range of CSR activities and, conversely, how to offer organizational CSR opportunities that appeal to employees’ diverse interest while ensuring organizational CSR efforts are strategically cohesive and appear genuine to employees. Future research will be required to develop this understanding.

With a peripheral strategy, a major hurdle for organizations is attracting employee participation in CSR initiatives, whereas the concern with an embedded strategy is getting employees to look beyond the confines of their job role. Therefore, employees with embedded CSR will require different performance management to those with peripheral CSR. This can be conceptualized as encouraging a progress versus commitment orientation; with an embedded CSR strategy rather than having a progress approach where employees are encouraged to achieve their goals as an end point, the organization should reinforce a commitment orientation where achieving their goals is seen as an indicator that they should do more. With a peripheral CSR strategy, because employees are coming from a low ‘base level’ of CSR a progress orientation, which enables employees to have explicit end goal achievement, is more likely to motivate them to engage in the initiatives that the organization offers.

### Limitations

As this research represents an initial exploration into employee perceptions and engagement in CSR behavior, there are some limitations that must be acknowledged. First, as we sought participants on a voluntary basis to talk about CSR, and further, because these participants were from organizations with relatively high CSR engagement, our sample may embody stronger CSR values than organizations without a strong CSR focus or employees who did not respond to the recruitment effort. This is somewhat mitigated by including participants in a broad range of positions within the NFP organization, and by including both employees that had and those that had not participated in corporate volunteering in the corporate sample. However, future research will need to investigate whether there is a marked difference in the way that employees in socially irresponsible organizations view and engage in CSR.

In a similar vein, participants were recruited specifically to discuss CSR, therefore social desirability bias may have been at play with participants wanting to emphasize their benefit to the environment or society at work. This could partially explain the search for meaning within the corporate sample as a defensive reaction to counteract the perception that they (or their organization) are not doing any good for society.

Finally, the salience of extra-role behavior in the three NFP participants who cited difficulty implementing environmental initiatives instead of in-role behavior could be due to the negative response they encountered rather than the importance and effort involved with the act.

Despite these limitations, we believe that the construct of eCSR is an important one as we move forward in our understanding of CSR more broadly. We have distinguished eCSR from other individual-level pro-social behaviors and have shown how employees from different contexts perceive CSR and their engagement in it differently. We hope that this research helps add cement to the micro-foundations of CSR by allowing us to see how employees make sense of CSR and broadening the implications for increasing eCSR engagement.

## Author Contributions

AS collected and analyzed data. KU framed introduction and discussion. Both authors edited the manuscript throughout the whole writing process.

## Conflict of Interest Statement

The authors declare that the research was conducted in the absence of any commercial or financial relationships that could be construed as a potential conflict of interest.

The reviewer OF and handling Editor declared their shared affiliation, and the handling Editor states that the process nevertheless met the standards of a fair and objective review.
